# Regression of liver fibrosis and hepatocellular carcinoma development after HCV eradication with oral antiviral agents

**DOI:** 10.1038/s41598-021-03272-1

**Published:** 2022-01-07

**Authors:** Hae Won Yoo, Jun Yong Park, Sang Gyune Kim, Young Kul Jung, Sae Hwan Lee, Moon Young Kim, Dae Won Jun, Jae Young Jang, Jin Woo Lee, Oh Sang Kwon

**Affiliations:** 1 Department of Internal Medicine, SoonChunHyang University School of Medicine, Digestive Disease Center and Research Institute, SoonChunHyang University Bucheon Hospital, 170 Jomaru-ro, Wonmi-gu, Bucheon, 14584 Korea; 2 Department of Internal Medicine, Yonsei University College of Medicine, Yonsei Liver Center, Severance Hospital, 50-1 Yonsei-ro, Seodaemun-gu, Seoul, 03722 Korea; 3 Korea University Ansan Hospital, Ansan, Korea; 4 Soonchunhyang University College of Medicine Cheonan Hospital, Cheonan, Korea; 5 Wonju Severance Christian Hospital, Wonju, Korea; 6 Hanyang University Hospital, Seoul, Korea; 7 Soonchunhyang University College of Medicine Seoul Hospital, Seoul, Korea; 8 Inha University Hospital, Incheon, Korea; 9 Department of Internal Medicine, Gil Medical Center, Gachon University College of Medicine, Incheon, Korea

**Keywords:** Hepatology, Liver fibrosis

## Abstract

We prospectively investigated the changes of liver stiffness (LS) and the occurrence of hepatocellular carcinoma (HCC) after hepatitis C virus (HCV) eradication using direct antiviral agents (DAA) over three years. LS measurement using transient elastography and serum fibrosis surrogate markers before treatment and at 48, 96, 144 weeks after starting direct-acting antivirals (DAA) according to the protocol were evaluated. Patients were also compared with historical cohort treated with pegylated interferon (peg-IFN). Sustained viral response (SVR) was observed in 95.8%. LS value in the patients achieving SVR significantly decreased over time (19.4 ± 12.9 kPa [baseline], 13.9 ± 9.1 kPa [48 weeks], 11.7 ± 8.2 kPa [96 weeks], 10.09 ± 6.23 [144 weeks], all *p* < 0.001). With matched analysis, the decrease in LS value was significantly larger in DAA group than peg-IFN group at both 48 weeks (29% vs. 9%) and 96 weeks (39% vs. 17%). The incidence of HCC was not significantly different between DAA and peg-IFN groups (5.5% vs. 5.4%) at 144 weeks. HCV eradication with DAA can lead to improvement of liver stiffness over time. The regression of fibrosis was greater in the group with DAA than peg-IFN.

Clinical trials registration: ClinicalTrials.gov (NCT02865369).

## Introduction

Hepatitis C virus (HCV) infection can lead to liver-related events (LRE) such as hepatocellular carcinoma (HCC), liver decompensation, and liver related death^1^. The achievement of sustained viral response (SVR) by HCV eradication can stop or reverse the damage of liver, thereby reducing the occurrence of liver related events^2^. The development of direct antiviral agents (DAA) has allowed over 90% of patients to reach SVR in clinical practice^3^.

After eradication of the virus, remaining liver fibrosis is the most predictive factor for the development of LRE. Regression of liver fibrosis after achieving SVR with pegylated interferon (peg-IFN) and ribavirin (RBV) combination treatment has been well established^4,5^. However, the degree of fibrosis improvement varies considerably from individual to individual. Especially in patients having advanced fibrosis or cirrhosis, morphological changes of the liver could be irreversible, meaning that they have residual risks for LRE^6^. As the progress of hepatic fibrosis is covert until the end stage of disease, a longitudinal assessment during follow-up is necessary for these patients^6^. However, long-term data regarding liver fibrosis improvement after SVR achievement through DAA and the degree of regression compared to peg-IFN/RBV are limited.

For assessing histologic status of liver, parenchymal biopsy was regarded as the ‘gold standard’ traditionally^7^. Liver biopsy entails the risk for sampling error and sometimes procedure-related adverse events such as bleeding, infection, bile leakage, and even death^8^. Thus, it is not appropriate for sequential assessment. To overcome these limitations, a non-invasive measurement for liver fibrosis has been developed. It has largely replaced invasive liver biopsy^9^. Serum markers including ‘aspartate aminotransferase-to-platelet ratio index’ (APRI) and ‘fibrosis index based on 4 factors’ (FIB-4) are two commonly used scoring systems for HCV^7^. Transient elastography (TE) using ultrasound based non-invasive method has been proven to be useful for measuring liver fibrosis^10^. It is now widely used in clinical practice due to its reliability and reproducibility^7^.

In this study, we aimed to evaluate long-term changes of liver stiffness using TE after achieving SVR through DAA treatment and examined how much HCC developed in relation to improvement of liver fibrosis.

## Subjects and methods

### Study design

This was a prospective, multi-center, observational study aimed at monitoring over five years. Patients were administered with 100 mg asunaprevir (ASV) twice per day plus 60 mg daclatasvir (DSV) once daily for 24 weeks. All patients underwent laboratory tests and transient elastography at baseline, 48 weeks (SVR24), 96 weeks (SVR72), and 144 weeks (SVR120). To compare the degree of fibrosis improvement with peg-IFN/RBV, a historical cohort was additionally analyzed as control.

### Patients

Patients with chronic hepatitis C and genotype 1b were screened for inclusion between August 2015 and December 2017. Inclusion criteria were: (1) age of 19 years and above; (2) chronically infected with HCV genotype 1b; (3) HCV RNA of more than 10,000 IU/mL; (4) patients believed to have significant fibrosis (defined as ≥ 6 kPa on TE); and (5) treatment-naïve or those who previously failed treatment with peg-IFN/RBV. Exclusion criteria were: (1) patients with baseline key NS5A RAVs (Y93 and/or L31); (2) estimated GFR < 30 mL/min; (3) elevated aspartate transaminase (AST) or alanine transaminase (ALT) of more than 100 IU/L; (4) coinfection with other hepatitis virus or human immunodeficiency virus (HIV); (5) decompensated liver disease or hepatocellular carcinoma; 6) liver or any other organ transplantation.

Medical records of peg-IFN + RBV treated patients were obtained retrospectively from HCV databases of two tertiary centers to do matching with patients receiving DAA treatment. From October 2010 to September 2014, patients with HCV genotype 1b infection and SVR achievement through peg-IFN + RBV regimen and available consecutive liver stiffness (LS) measurement were collected.

All patients underwent DCV/ASV and provided written informed consent. This study was approved by the Institutional Review board (IRB) of each participating center (IRB number; SCHBC 2016-06-014-020) and registered at ClinicalTrials.gov (NCT02865369, First registration: 12/08/2016, https://clinicaltrials.gov/ct2/show/NCT02865369?cond=NCT02865369&draw=2&rank=1).

### Liver stiffness measurement

TE was performed with a FibroScan (Echosens, Paris, France) using M and XL probes. Experienced physicians performed all examinations. Liver stiffness was assessed through the intercostal space with patients in the supine position and right arm above the head during breath hold. The value of LS was considered reliable when 10 validated measurements were acquired with a success rate of at least 60% and interquartile range (IQR) < 30% of the median according to current guidelines^10^. Stages of liver fibrosis were categorized according to LS value: F0–F1 < 6 kPa; 6 kPa ≤ F2 < 8 kPa; 8 kPa ≤ F3 < 12 kPa; and F4 ≥ 12 kPa. The classification was based on cut-off values of each stage of previous reports^11–13^.

### Clinical and laboratory assessment

At enrollment, baseline data including age, sex, alcohol intake, body mass index (BMI), and presence of comorbidity were collected. Laboratory data were taken at baseline and at 48 weeks, 96 weeks, and each year thereafter according to the study protocol. APRI^14^ and FIB-4^15^ scores were calculated using laboratory data and age. For the surveillance of HCC, all patients underwent alpha-fetoprotein test and ultrasonography every six months. If necessary, computed tomography and/or magnetic resonance imaging was performed to confirm HCC.

### Outcomes

Primary outcomes were changes in LS value at 48 weeks (SVR24), 96 weeks (SVR72), and 144 weeks (SVR120) after initiation of DAA and changes in LS value from 48 weeks (SVR24) at each stage. Secondary outcomes were alteration of serum fibrosis markers (APRI score and FIB-4 index), occurrence of HCC after DAA treatment, factors associated with significant regression, and changes of LS compared with peg-IFN/RBV treated group. Significant regression was defined as more than 30% reduction of liver stiffness from baseline LS value.

To assess the extent of fibrosis regression after achievement of SVR, additional analyses were performed with propensity matching. The degree of improvement in liver stiffness was compared between peg-IFN/RBV-treated and DAA-treated patients after covariate balance with propensity score matching and inverse probability of treatment weighting using propensity score.

### Sample size calculation

The reference value for the improvement rate of the fibrosis stage according to liver stiffness was obtained from poster 777 presented at 2015 AASLD (American Association for the Study of Liver Disease) which evaluated 137 participants of sustained virologic responders at 24 weeks after LDV/SOF-based therapy. They showed 80% decline in liver stiffness assessed by TE at 24 weeks after the therapy (improved in 39%, stable in 60%, and worsened in 0.7% compared to the baseline)^16^.

For the improvement of fibrosis stage based on the liver stiffness measurement at 48 weeks after treatment termination as the primary endpoint, 103 subjects would be needed to produce a two-sided 95% confidence interval with a width equal to 0.2 when the estimated improvement was assumed to be 0.39. This calculation assumed a dropout rate of 10% using one proportion confidence interval formula based on a simple asymptotic distribution (92/0.9 = 102.22).

### Statistical analyses

All statistical analyses were performed using R statistics. Continuous variables are presented as mean ± standard deviation and categorical variables are presented as absolute number (percentage). Multiple paired data at each time point (at baseline, 48 weeks, 96 weeks and 144 weeks) were compared using K-paired sample Friedman tests. For comparing groups, *p* values were computed by Chi-square test for categorical variables and t-test for continuous variables. Binary Logistic regression was used to identify independent variables associated with significant regression. To reduce the bias between peg-IFN + RBV and DCA + ASV groups, propensity score matching (PSM) was used. PSM was performed at a 1:1 ratio with the initial cohort using nearest neighbor matching and a caliper of 1.5. Matching was performed using two manners. For PSM model 1 [PS1], matching variables included age, status of liver disease (chronic liver disease or cirrhosis), AST, ALT, and platelet count. For PSM model 2, baseline liver stiffness was used for matching. Standardized mean differences (SMDs) were used to assess the balance between the two groups after PSM. Matched pairs were compared using McNemar test for categorical variables and paired t-test for continuous variables. Statistical significance was defined at *p* < 0.05. R version 3.6.3 was used for all statistical analyses^17^.

## Results

### Characteristics of patients

**Figure 1:**
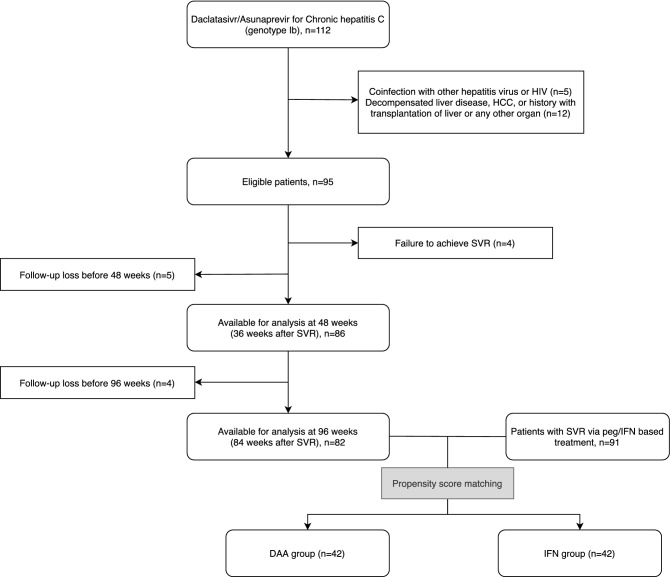
Study design and selection of patients.

A total of 112 patients were screened and 17 patients who did not meet the criteria were excluded as shown in Fig. 1. Baseline characteristics of patients are summarized in Table 1. The mean age was 66.1 ± 9.8 years. There were 48 (50.5%) males. Ninety-one (95.8%) patients achieved SVR. Thirty-eight (40.0%) patients had compensated LC at baseline. Thirty (31.6%) patients had a history of previously treated peg-IFN/RBV. Mean value of LS was 18.3 ± 12.6 kPa. More than half of patients showed LS value over 12 kPa at baseline. On the basis of baseline LS value, distributions of estimated fibrosis stages F2, F3, and F4 were 11.0%, 31.9%, and 57.1%, respectively.

**Table 1:** Baseline characteristics of patients.

Variable†	n = 95
Age, year	66.1 ± 9.8
Sex, male	48 (50.5)
BMI, kg/m^2^	24.46 ± 3.17
**Baseline liver status**	
Chronic liver disease	57 (60.0)
Compensated LC	38 (40.0)
Previous pegIFN/RBV	30 (31.6)
Liver stiffness, kPa	18.26 ± 12.63
**Fibrosis stage**	
F2	10 (11.0)
F3	29 (31.9)
F4	52 (57.1)
AST	60.0 ± 23.3
ALT	41.7 ± 22.8
Total bilirubin, μMol/L	1.00 ± 0.59
Platelet, /μL	140.4 ± 52.2
PT, INR	1.09 ± 0.19
Albumin, g/dL	4.01 ± 0.46

^†^Continuous variables are described as mean ± standard deviation. Nominal variables are described as number (percentage).

BMI, body mass index; LC, liver cirrhosis; IFN, interferon; AST, aspartate transaminase; ALT, alanine transaminase; PT, prothrombin time; INR, internationalized ratio; SVR, sustained viral response.

### Changes of liver fibrosis at 48, 96, and 144 weeks

**Figure 2:**
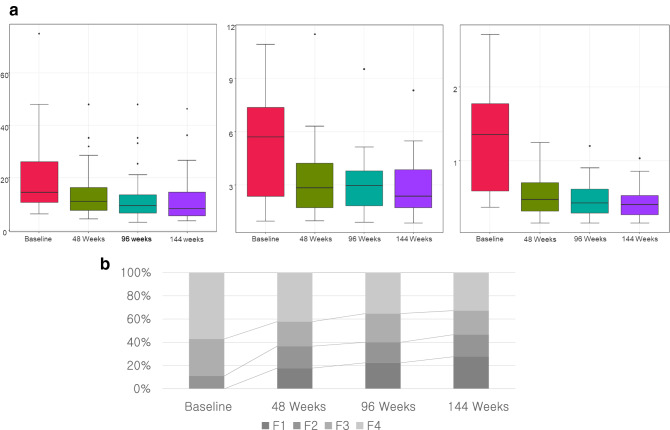
(**a**) Comparison of changes in liver stiffness, APRI, and FIB-4 index. (**b**) Distribution of fibrosis stage across different time points.

At 48 weeks during follow-up, 86 patients were available for analysis. The overall LS significantly decreased after DAA treatment from 19.37 ± 12.86 kPa at baseline to 13.9 ± 9.1 kPa at 48 weeks (Fig. 2a, Table 2). Other estimated stages of fibrosis also showed improvement (Fig. 2b). Among patients with estimated F4 at baseline (n = 52), 17 (32.7%) showed at least one stage of decline in fibrosis stage at 48 weeks after DSV/ASV administration. Serum fibrosis markers were significantly improved after SVR. Similar to TE, APRI score decreased from 1.36 ± 0.86 to 0.66 ± 0.62 (*p* < 0.001) and FIB-4 decreased from 6.29 ± 4.67 to 3.25 ± 2.14 (*p* < 0. 001) (Fig. 2a, Table 2).

**Table 2:** Changes in liver fibrosis markers after DAA treatment.

	Baseline	48 W	96 W	144 W	*p*-value^†^
Liver stiffness, kPa	19.37 ± 12.86	13.88 ± 9.13	11.70 ± 8.23	10.09 ± 6.23	< 0.001
FIB-4	6.29 ± 4.67	3.25 ± 2.14	2.87 ± 1.59	2.89 ± 1.70	< 0.001
APRI	1.36 ± 0.86	0.66 ± 0.62	0.50 ± 0.29	0.43 ± 0.21	< 0.001

^†^K-paired sample Friedman tests were used as normality tests were not satisfied.

DAA, direct antiviral agent; FIB-4, fibrosis-4; APRI, Aspartate transaminase to platelet ratio index; W, week.

Eighty-two patients were available for analysis at 96 weeks. LS further decreased to 11.7 ± 8.2 kPa from 13.9 ± 9.1 kPa at 48 weeks (*p* < 0.001). The proportion of non-F4 patients was additionally increased as shown in Fig. 2b. Twenty-three (44.2%) with F4 at baseline showed regression for fibrosis stage at 96 weeks. Both APRI score and FIB-4 index showed significant decreases compared to those at baseline (0.50 ± 0.29 vs. 1.36 ± 0.86 and 2.87 ± 1.59 vs. 6.29 ± 4.67, both *p* < 0.001). APRI score showed significant reductions at 96 weeks compared to the value at 48 weeks (*p* < 0.001) (Table 2).

For 58 patients with available LS measurement during a long-term follow-up, mean LS value of 144 weeks was 10.71 ± 7.80, which was a further improvement compared to that at 96 weeks. Of 37 patients showing F4 stage at baseline, 19 (51.3%) no longer had cirrhosis at 144 weeks (Fig. 2). Similar to LS value, APRI score showed significant decrease from 96 and 48 weeks (both p < 0.001).

### Factors associated with regression of fibrosis stage

Significant fibrosis regression was observed in 41 (47.7%) of 86 and 52 (63.4%) of 82 patients at 48 and 96 weeks, respectively. In patients with compensated cirrhosis, 17 (44.7%) and 24 (63.2%) of 38 patients showed significant regression at 48 and 96 weeks, respectively. Univariate logistic regression analyses were performed to find factors related to significant regression. However, no factor showed significant association with fibrosis regression at 48 weeks or 96 weeks (Table S1).

### Comparison of fibrosis regression with IFN-treated group

Of a total of 92 patients treated with pegIFN/RBV who had LS results, 42 patients were compared as matched pairs according to PS1. Baseline characteristics before and after propensity score matching are shown in Table S2. There were no significant differences in characteristics of patients except for platelet count after matching. The DAA group showed 27% reduction from index LS value whereas the peg-IFN group showed 5% reduction. The proportional change was also greater in the DAA group after matching (−29% vs. −9%, *p* < 0.0001) (Table 3). Significant regression (> 30% reduction in liver stiffness) was observed frequently in the DAA group before and after matching both at 48 and 96 weeks. At 48 weeks, the DAA group showed a reduction in stiffness (5.74 ± 8.53 kPa) compared to the peg-IFN group (0.75 ± 1.68, *p* = 0.0004) after matching. At 96 weeks, both peg-IFN group and DAA group showed further decrease in LS value, with the magnitude of decrease being greater in the DAA group (7.11 ± 8.44 kPa vs. 1.55 ± 2.85 kPa) (Table 3).

**Table 3:** Changes in liver stiffness before and after propensity score matching (PS1).

	Before PS matching			After PS matching		
Outcome	IFN (n = 92)	DAA (n = 82)	p value	IFN (n = 42)	DAA (n = 42)	p value
Change of LS value^†^ at 48 W, kPa (%)	0.62 ± 1.39 (8%)	5.78 ± 7.69 (27%)	< 0.0001	0.75 ± 1.68 (9%)	5.74 ± 8.53 (29%)	< 0.0001
Change of LS value at 96 W, kPa (%)	1.46 ± 2.79 (15%)	7.92 ± 8.28 (37%)	< 0.0001	1.55 ± 2.85 (17%)	7.11 ± 8.44 (39%)	0.0001
Significant regression^‡^ at 48 W	5 (5.5)	40 (48.8)	< 0.0001	3 (7.1)	23 (54.8)	< 0.0001
Significant regression at 96 W	12 (13.2)	52 (63.4)	< 0.0001	5 (15.6)	27 (64.3)	< 0.0001

^†^Changes of LS value are described as subtracted change from baseline LS (percentage change).

^‡^Significant regression was defined as more than 30% reduction of liver stiffness from baseline.

PS, propensity matching; IFN, interferon; DAA, direct antiviral agents; LS, liver stiffness; W, week.

To exclude the bias derived from differences in baseline liver stiffness between two group, matching using the liver stiffness [PS2] was performed with 35 pairs (Table S2). DAA group also showed significant reduction in liver stiffness at 48 weeks (13.93 ± 17.58 vs. 2.22 ± 2.22, p = 0.0004).

### HCC occurrence

No patient developed HCC at 48 weeks after starting DAA. Three (6.1%) patients developed HCC at 96 weeks in the DAA group. At peg-IFN group, 3 (3.3%) of 91 showed HCC at 96 weeks. At 144 weeks, HCC occurred in 5 (5.5%) patients of the DAA group and 5 (5.4%) patients of the peg-IFN group, showing no significant difference between the two groups. Serial LS values of patients with HCC occurrence on the DAA group are summarized in Table 4.

**Table 4:** Characteristics of patients with HCC development.

Patients No	Sex/age	HCC occurrence	Baseline LS	LS at 48 week	LS at 96 week	LS at 144 week
1	M/59	96 weeks	38.0	28.4 (− 25.3%)	26.7 (−29.7%)	
2	F/76	96 weeks	32.1	25.1 (− 21.8%)	35.0 (+ 0.09%)	
3	M/69	96 weeks	45.0	39.0 (−13.3%)	13.4 (−70.2%)	
4	F/75	144 weeks	25.7	20.0 (−22.2%)	14.5 (−43.6%)	19.8
5	M/63	144 weeks	17.6	12.0 (−31.8%)	9.7 (−44.9%)	10.5

HCC, hepatocellular carcinoma; LS, liver stiffness.

## Discussion

In the era of IFN based regimen, main concerns for HCV treatment were increased SVR and monitoring of liver-related events for non-virologic responders. The development of IFN-free regimen has made it easier to achieve SVR with a simplified method having a good safety profile^18^. Hence, the focus has shifted to the improvement in hepatic fibrosis after viral response, which could help us figure out who will have the remained risk^19^. Yet, prospective data regarding the long-term outcome and the potential fibrosis regression after DAA treatment are insufficient.

Another important issue associated with DAA treatment is whether more HCC occurs after SVR induced by DAA^20,21^. Recent studies have suggested that SVR by DAA can lower the risk for HCC development^22,23^. In addition, pre-DAA albumin, post-DAA LS value, and post-DAA albumin are independent predictors for HCC development^18^.

Main findings of the present study were: (1) regression of fibrosis continued up to 144 weeks after starting DCV/ASV: (2) regression of fibrosis by more than 30% was observed in 47.7% and 63.4% of patients at 48 and 96 week from the initiation of DAA; (3) the degree of fibrotic change was consistently larger in the DAA group than in the peg-IFN/RBV group before and after PSM; and (4) both groups showed similar HCC occurrence after SVR until 144 weeks.

Several studies have evaluated a change of liver fibrosis after IFN based regimen using paired biopsies. Lower baseline fibrotic stage, younger age, lower viral load, lower BMI, and higher baseline ALT level have been suggested as factors associated with larger improvement in liver histology^4,5^. In a meta-analysis with three randomized control trials, about one third of cirrhotic patients showed decrease in fibrosis score at 24 weeks after the end of treatment^4^. In another study, the reversal of cirrhosis was observed in 75 (49%) of 153 patients^5^.

This study showed results consistent with previous studies on DAA treatment. They reported that significant regression was observed in 40–46% at post-SVR12. In addition, about 31–38% of cirrhotic patients according to LS value before treatment were re-categorized as non-cirrhotic after SVR^24–27^.

We followed up LS measurement and serum markers for a longer duration compared to previous studies. The LS value showed significant reductions at 3 years even after starting the treatment. A recent study on HIV/HCV coinfected patients has reported that LS measured using TE shows a gradual decline at about 4 years after starting the treatment^28^. Another study with long-term LS measurement assessment for 5 years has reported that LS value shows a plateau decline until 5 years for patients after receiving IFN with DAA treatment^29^. When further long-term data of our cohort become available, they could help us determine potential markers for fibrosis regression.

Although TE is designed to assess the degree of liver fibrosis, it is also influenced by hepatic inflammation^30^. Thus, the comparison the LS from baseline may show a mixed effect of the improvement both in liver fibrosis and inflammation. To overcome this issue, the statistical analysis was performed to compare the difference of the LS value at 96 weeks and 144 weeks from 48 weeks (SVR24). The LS value showed significant regression in comparison between any time period (48 weeks vs. 96 weeks, 96 weeks vs. 144 weeks, 48 weeks vs. 144 weeks, all p < 0.001). Therefore, HCV eradication using DAA could bring about improvement not only in hepatic inflammation but also in fibrosis.

To the best of our knowledge, this is the first study to compare the extent of fibrosis improvement between DAA and peg-IFN + RBV regimen using matched analysis. However, there were incurable imbalances in cohorts between the two groups. The major difference within cohorts was attributed to a broader therapeutic window for HCV treatment in the DAA group. We acknowledge that there are discrepancies even after matching, especially on platelet count. However, according to previous study^24^, lower platelet count is associated with poor improvement of liver fibrosis. In our study, rather, DAA group with relatively lower platelet count showed better outcome on fibrosis improvement compared to peg-IFN/RBV group with higher platelet count. For assessing the degree of regression accurately, we used two models for matching and we evaluated a fibrotic alteration as a gap of the LS value and a proportional change. The DAA group showed larger reduction consistently. Moreover, earlier reduction was observed on DAA group. In a previous meta-analysis^31^, the absolute decrease in LS between before and at the end of the treatment was greater in DAA group than in peg-IFN/RBV group. Considering that cirrhosis was more common in DAA treated patients in this meta-analysis, a proportional change of LS value rather than an absolute difference from baseline would be more appropriate to compare. They suggested that the larger magnitude of decline in LS might have resulted from characteristics of DAA, which can eliminate virus more quickly, leading to rapid declines of inflammation and fibrogenesis. Accumulation of robust evidence is necessary to support this assumption.

Several previous studies have demonstrated that higher LS value, alcohol intake, type 2 diabetes, and absence of thrombocytopenia could be factors associating with significant regression^24,32,33^. In our study, however, neither baseline LS value, alcohol intake, type 2 diabetes, nor thrombocytopenia was related to a significant regression. However, suggested factors were quite different among various studies. This should be clarified with further studies.

This study has some limitations. In 2015, when this study was planned and started, the only available DAA was DCV/ASV. Now in the era of pan-genomic DAA, DCV/ASV is no longer a preferred option for HCV treatment. However, the major concern of this study is how much liver stiffness has improved since the use of DAA, not specific drugs. Further prospective studies including various widely used regimens are required. Even we calculated sample size from previous study, there could be a risk of beta error due to its relatively small sample size. Even the treatment with DAA showed longitudinal improvement of liver fibrosis, the caution is need in interpreting the results of this study. Also, included patients of our study were restricted to genotype 1b infection. Although a previous study with a small number of subjects has shown that significant fibrosis regression after DAA is associated with genotype 1, it is currently unclear whether the improvement in liver fibrosis is associated with viral genotype^32^. Recent studies which investigated the alteration of liver stiffness after DAA treatment, there was a rapid decrease in LS within a few weeks after DAA treatment^28,32^. We did not check LS at the end of DAA treatment because it was assumed that fibrosis regression occurred gradually over time rather than immediately after drug use. Although many studies have demonstrated that liver stiffness measured by TE is well correlated with fibrosis stage, suggested cut-off values have discrepancies according to studies^10^. Thus, the suggested liver fibrosis stage used in our study requires attention in its interpretation. In addition, the number of cohort was not large enough to evaluate the association between the change of liver stiffness and predictive factors. Finally, this study was not a controlled trial between DAA and peg-IFN based regimen because a direct comparison was not feasible ethically or time wise. Recently, there has been an increased interest in the risk of HCC development after DAA treatment^19,34^. Although the number of HCC occurrence in this study was too small to compare between two groups, the incidence of HCC was not significantly different from that in the peg-IFN/RBV group. Furthermore, it was not sufficient for identifying a relationship with the extent of regression.

In conclusion, eradication of HCV with DCV/ASV resulted in a continuous improvement of liver stiffness over time. SVR induced by DAA treatment was strongly associated with a greater regression of fibrosis. DAA resulted in a comparable occurrence of HCC to peg-IFN/RBV treatment. Further studies are needed to confirm our results and identify the group of patients who need careful monitoring after SVR.

### Ethics approval and consent to participate

The study protocol was approved by the Institutional Review Board of each hospital (IRB number; SCHBC 2016-06-014-020). The study protocol conformed to ethical guidelines of the World Medical Association’s Declaration of Helsinki. All participants provided written informed consent.

## Data availability

The data that support the findings of this study are available on request from the corresponding author.

## Supplementary Information

Supplementary Information.

## Abbreviations

HCV: Hepatitis C virus
LRE: Liver related events
HCC: Hepatocelluar carcinoma
SVR: Sustained viral response
DAA: Direct antiviral angents
peg IFN: Peglyated interferon
RBV: RibavirinRBV/Ribavirin
APRI: Aspartate aminotransferase to platelet ratio index
FIB 4: Fibrosis index based on 4 factors
TE: Transient elatography
D CV: Daclatasvir
A SV: Asunaprevir
AST: Aspartate transaminase
ALT: Alanine transaminase
HIV: Human immunodeficiency virus
LS: Liver stiffness
PSM: Propensity score matching
SMD: Standardized mean difference
