# Supplementary material for: Regression of liver fibrosis and hepatocellular carcinoma development after HCV eradication with oral antiviral agents
Source: Sci Rep. 2022 Jan 7;12:193. doi: 10.1038/s41598-021-03272-1 (PMC8742091; doi:10.1038/s41598-021-03272-1)
Supplement: Supplementary file 1 — Supplementary Information. [file 41598_2021_3272_MOESM1_ESM.docx]

**Table S1. Factors associated with improvement in fibrosis stage at 48 and 96 weeks after starting DAA treatment**

|  | **48 weeks** | | **96 weeks** | |
| --- | --- | --- | --- | --- |
| Variable | Univariate analysis | | Univariate analysis | |
|  | Odd ratio (95% CI) | *p*-value | Odd ratio (95% CI) | *p*-value |
| Compensated LC (vs. CLD) | 0.885 (0.376 – 2.083) | 0.780 | 1.286 (0.517 – 3.199) | 0.589 |
| Age | 0.969 (0.928 – 1.013) | 0.166 | 0.959 (0.913 – 1.007) | 0.093 |
| Female (vs. male) | 1.211 (0.519 – 2.825) | 0.659 | 1.000 (0.407 – 2.456) | 1.000 |
| HTN | 0.793 (0.250 – 2.517) | 0.6937 | 0.429 (0.129 – 1.423) | 0.166 |
| DM | 1.371 (0.385 – 4.886) | 0.626 | 0.652 (0.181 – 2.352) | 0.514 |
| BMI | 0.972 (0.867 – 1.116) | 0.689 | 1.007 (0.872 – 1.163) | 0.924 |
| Alcohol | 0.809 (0.170 – 3.853) | 0.790 | 1.167 (0.201 – 6.783) | 0.864 |
| History. of IFN based regimen | 0.484 (0.191 – 1.226) | 0.126 | 0.608 (0.236 – 1.565) | 0.302 |
| AST | 1.005 (0.988 – 1.024) | 0.558 | 1.006 (0.987 – 1.026) | 0.522 |
| ALT | 1.011 (0.992 – 1.031) | 0.264 | 1.020 (0.997 – 1.043) | 0.095 |
| Baseline LS | 1.031 (0.994 – 1.069) | 0.103 | 1.042 (0.996 – 1.090) | 0.072 |
| Platelet | 1.000 (0.992 – 1.009) | 0.925 | 0.998 (0.990 – 1.007) | 0.709 |
| Albumin | 1.116 (0.437 – 2.850) | 0.818 | 0.599 (0.212 – 1.696) | 0.335 |
| Total bilirubin | 1.100 (0.544 – 2.225) | 0.791 | 2.713 (0.840 – 8.769) | 0.095 |
| INR | 0.309 (0.017 – 5.534) | 0.425 | 17.365 (0.145 – 2086.747) | 0.243 |

LC, liver cirrhosis; CLD, chronic liver disease; HTN, hypertension; DM, diabetes mellitus; BMI, body mass index; IFN, interferon; AST, aspartate transaminase; ALT, alanine transaminase; LS, liver stiffness; INR, international normalized ratio

**Table S2. Baseline characteristics of study patients before and after propensity score analysis**

|  |  | **Before PS matching** | | | | **After PS matching** | | | |
| --- | --- | --- | --- | --- | --- | --- | --- | --- | --- |
| PS1 | Variable | IFN (n=91) | DAA (n=82) | *p* value | SMD | IFN (n=42) | DAA (n=42) | *p* value | SMD |
|  | Liver cirrhosis | 3 (3.3) | 35 (43.2) | <0.001 | 1.072 | 3 (7.1) | 3 (7.1) | 1.000 | <0.001 |
|  | AST | 54.0±37.9 | 60.8±23.8 | 0.151 | 0.214 | 49.4±40.1 | 58.4±24.7 | 0.216 | 0.270 |
|  | ALT | 66.0±58.1 | 39.5±21.3 | <0.001 | 0.604 | 47.9±46.7 | 39.3±20.0 | 0.255 | 0.239 |
|  | PLT | 201.7±60.5 | 137.0±53.9 | <0.001 | 1.130 | 176.1±46.1 | 150.1±57.9 | 0.001 | 0.497 |
|  | Total bilirubin | 0.82±0.35 | 1.01±0.62 | 0.017 | 0.376 | 0.83±0.44 | 0.93±0.48 | 0.347 | 0.211 |
| PS2 | Variable | IFN (n=91) | DAA (n=82) | *p* value | SMD | IFN (n=35) | DAA (n=35) | *p* value | SMD |
|  | LS | 6.51±2.76 | 19.10±12.75 |  | 1.365 | 9.00±2.82 | 9.57±1.99 | 0.3310 | 0.234 |

PS, propensity score; PS1, propensity score matching model 1; IFN, interferon; DAA, direct antiviral agents; SMD, standardized mean difference; AST, aspartate transaminase; ALT, alanine transaminase; PLT, platelet; PS2, propensity score matching model 2; LS, liver stiffness

**Table S3. Changes in liver stiffness before and after propensity matching (PS2)**

|  | **Before PS matching** | | | **After PS matching** | | |
| --- | --- | --- | --- | --- | --- | --- |
| Outcome | IFN (n=92) | DAA (n=82) | *p* value | IFN (n=35) | DAA (n=35) | *p* value |
| Change of LS value^†^ at 48W, kPa (%) | 0.62 ± 1.39 (8%) | 5.78 ± 7.69 (27%) | <0.0001 | 2.22 ± 2.22 | 13.93 ± 17.58 | <0.001 |
| Change of LS value at 96W, kPa (%) | 1.46 ± 2.79 (15%) | 7.92 ± 8.28 (37%) | <0.0001 | 31.06 ± 21.90 | 33.83 ± 30.96 | 0.570 |

*† Changes of LS value are described as subtracted change from baseline LS (percentage change)*

*PS, propensity matching; IFN, interferon; DAA, direct antiviral agents; LS, liver stiffness; W, week*
